# Diaphragmatic pacing for the prevention of sudden unexpected death in epilepsy

**DOI:** 10.1093/braincomms/fcac232

**Published:** 2022-09-16

**Authors:** Benton S Purnell, Alexander Braun, Denise Fedele, Madhuvika Murugan, Detlev Boison

**Affiliations:** Department of Neurosurgery, Robert Wood Johnson Medical School, Rutgers University, 10 Plum St., New Brunswick, NJ 08901, USA; Department of Neurosurgery, Robert Wood Johnson Medical School, Rutgers University, 10 Plum St., New Brunswick, NJ 08901, USA; Department of Neurosurgery, Robert Wood Johnson Medical School, Rutgers University, 10 Plum St., New Brunswick, NJ 08901, USA; Department of Neurosurgery, Robert Wood Johnson Medical School, Rutgers University, 10 Plum St., New Brunswick, NJ 08901, USA; Department of Neurosurgery, Robert Wood Johnson Medical School, Rutgers University, 10 Plum St., New Brunswick, NJ 08901, USA; Brain Health Institute, Rutgers University, 683 Hoes Lane West, Piscataway, NJ 08854, USA

**Keywords:** epilepsy, SUDEP, respiratory arrest, diaphragmatic pacing, electrophrenic respiration

## Abstract

Sudden unexpected death in epilepsy is the leading cause of epilepsy related death. Currently, there are no reliable methods for preventing sudden unexpected death in epilepsy. The precise pathophysiology of sudden unexpected death in epilepsy is unclear; however, convergent lines of evidence suggest that seizure-induced respiratory arrest plays a central role. It is generally agreed that sudden unexpected death in epilepsy could be averted if the patient could be rapidly ventilated following the seizure. The diaphragm is a muscle in the chest which contracts to draw air into the lungs. Diaphragmatic pacing is a surgical intervention which facilitates normal ventilation in situations, such as spinal cord injury and sleep apnoea, in which endogenous respiration would be inadequate or non-existent. In diaphragmatic pacing, electrodes are implanted directly onto diaphragm or adjacent to the phrenic nerves which innervate the diaphragm. These electrodes are then rhythmically stimulated, thereby eliciting contractions of the diaphragm which emulate endogenous breathing. The goal of this study was to test the hypothesis that seizure-induced respiratory arrest and death can be prevented with diaphragmatic pacing. Our approach was to induce respiratory arrest using maximal electroshock seizures in adult, male, C57BL6 mice outfitted with EEG and diaphragmatic electrodes (*n* = 8 mice). In the experimental group, the diaphragm was stimulated to exogenously induce breathing. In the control group, no stimulation was applied. Breathing and cortical electrographic activity were monitored using whole body plethysmography and EEG, respectively. A majority of the animals that did not receive the diaphragmatic pacing intervention died of seizure-induced respiratory arrest. Conversely, none of the animals that received the diaphragmatic pacing intervention died. Diaphragmatic pacing improved postictal respiratory outcomes (two-way ANOVA, *P* < 0.001) and reduced the likelyhood of seizure-induced death (Fisher’s exact test, *P* = 0.026). Unexpectedly, diaphragmatic pacing did not instantly restore breathing during the postictal period, potentially indicating peripheral airway occlusion by laryngospasm. All diaphragmatically paced animals breathed at some point during the pacing stimulation. Two animals took their first breath prior to the onset of pacing and some animals had significant apnoeas after the pacing stimulation. Sudden unexpected death in epilepsy results in more years of potential life lost than any other neurological condition with the exception of stroke. By demonstrating that seizure-induced respiratory arrest can be prevented by transient diaphragmatic pacing in animal models we hope to inform the development of closed-loop systems capable of detecting and preventing sudden unexpected death in epilepsy.

## Teaser

Respiratory arrest is a crucial feature of the pathophysiology of sudden unexpected death in epilepsy (SUDEP), an enigmatic consequence of convulsive seizures. Purnell *et al*. report that diaphragmatic pacing can prevent seizure-induced respiratory arrest in a mouse model and discuss potential implications for the prevention of SUDEP.

## Graphical Abstract

**Graphical abstract:**
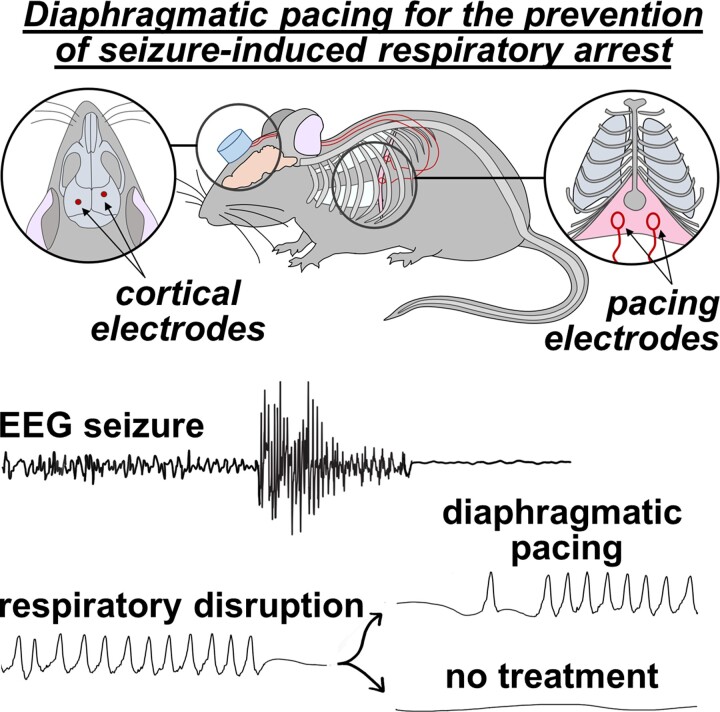

## Introduction

Sudden unexpected death in epilepsy (SUDEP) is the leading cause of premature death in patients with refractory epilepsy.^1^ Among neurological conditions, SUDEP results in more years of potential life lost than any other with the exception of stroke.^2^ Convergent lines of evidence from epilepsy patients and animal models of seizure-induced death implicate respiratory failure as a critical contributor to the mortality seen in SUDEP.^3,4^ The precise cause of the respiratory arrest seen in SUDEP has been the subject of intense empirical investigation. Candidate mechanisms include postictal respiratory instability due to serotonergic/noradrenergic dysfunction, excessive adenosinergic inhibition of the brainstem, inappropriate activation of the mammalian diving reflex, and propagation of spreading depolarization waves into the brainstem.^5,6,7,8,9^ Despite mechanistic insights into its pathophysiology, there are currently no interventions capable of reliably preventing SUDEP.^4^ It has been observed in epilepsy monitoring units that SUDEP can be prevented if resuscitative efforts are initiated within the first three minutes of respiratory arrest.^3^ This indicates that SUDEP is due to a disruption in respiratory function which is transient, but of sufficient duration to be fatal, as opposed to permanent damage to the respiratory system.^4^ Thus, if patients could be artificially ventilated during the seizure-induced apnoea, it is likely that death could be prevented. Unfortunately, ventilatory assistance is not a practical prevention strategy because SUDEP typically occurs without warning, during the night, while the patient is alone, making it highly improbable that a person with the requisite training and equipment would be available immediately.^10^

The diaphragm is a muscle located at the base of the chest which contracts to draw air into the lungs.^11^ Diaphragmatic pacing is a FDA approved surgical intervention which facilitates breathing in situations in which endogenous respiration would be inadequate or non-existent.^12^ In diaphragmatic pacing, electrodes are implanted directly onto the diaphragm or adjacent to the phrenic nerves which innervate the diaphragm. These electrodes are rhythmically stimulated to elicit contractions which emulate normal breathing.^12^ Diaphragmatic pacing has been used to treat the respiratory disruption associated with disorders such as spinal cord injury, central hypoventilation syndrome, amyotrophic lateral sclerosis, and central sleep apnoea.^13,14,15,16,17^ Although it is has been used in humans for more than a century, stimulation of the diaphragm to induce breathing has never been used in the context of epilepsy.^2–8^ A serious drawback of diaphragmatic pacing is that chronic continuous stimulation can lead to fatigue of the nerve or the muscle.^19^ This would not be an issue if pacing only needed to be applied intermittently.^19^

The goal of this study was to determine whether diaphragmatic pacing is capable of preventing seizure-induced respiratory arrest and death following maximal electroshock seizures in mice. The development of an implantable closed-loop diaphragmatic pacing system capable of detecting respiratory arrest and stimulating to restore breathing when needed may enable the prevention of SUDEP.

## Materials and methods

### Animals

The adult (>10-week-old) male C57BL6 mice used in this study were bred on-site in a Rutgers University Comparative Medicine Resources vivarium and not used in any prior procedures. The mice were housed under normal 12:12 LD conditions (lights on at 0600, lights off at 1800). Mice had ad libitum access to food (PicoLab 5058; LabDiet, St. Louis, MO) and water. All procedures and protocols used in this study were approved by the Rutgers University Institutional Animal Care and Use Committee in accordance with the international guidelines set by the Association for Assessment and Accreditation of Laboratory Animal Care. Care was taken to use the minimum number of animals possible and to minimize their pain and distress.

### Surgical instrumentation

The diaphragmatic electrodes were prepared prior to the surgery. Two 10 cm segments of wire electrode (AS632; Cooner Wire, Chatsworth, CA) were cut and stripped of 2 cm of insulation on one end and ∼0.3 cm on the other. The tip of each wire missing 2 cm of insulation was wrapped around itself at the junction between the insulated and uninsulated wire (2 cm away from the tip) forming a roughly circular loop. The wrapped segment of wire was brushed with adhesive to secure it in position (PT-33; Robart, Batavia, IL). On the other end of each wire, the tip missing ∼0.3 cm of insulation was soldered to a female gold-plated pin (363/0; Plastics One, Roanoke, VA). The net result is two ∼8 cm segments of insulated wire with a female gold-plated pin on one end and a roughly circular uninsulated wire loop on the other end (2 cm in circumference, and 0.64 cm in diameter). During the surgical procedure described below, each of the two uninsulated wire loop ends are affixed against each hemidiaphragm. The other end of each wire will emerge from an incision in the body wall, be tunnelled under the skin to an incision in the scalp, and integrated into a headmount. The ∼8 cm length of each wire is unnecessarily long in relation to the distance between the diaphragm and the headmount on a typical mouse; however, this excess slack makes it easier to get the headmount end of the wire tunnelled under the skin and integrated into the pedestal without tugging or applying any pressure to the diaphragm end which risks disturbing the position of the electrode.

Mice in the experimental and control groups underwent the same surgical instrumentation. The mice were anaesthetized with isoflurane (1–3% in 100% O_2_) which was maintained for the duration of the surgery. Electric shears were used to remove the fur on the scalp and the ventral abdomen approximately 2 cm above and below the bottom of the ribcage. Ointment was applied to the eyes to prevent corneal drying (Lubrifresh P.M.; Major Pharmaceuticals, Livonia, MI). The animal was secured onto a sterile surgical field on a warming pad (PhysioSuite; Kent Scientific, Torrington, CT) in a supine position. The exposed skin on the abdomen was disinfected with a povidone-iodine solution and 70% ethanol. Bupivacaine was injected along the site of the intended incision (0.1 mL, 0.25%; Sigma-Aldrich, St. Louis, MO). A transverse incision was made in the skin across the ventral abdomen just caudal to the bottom of the ribcage. A transverse incision was made through the body wall and peritoneum just below the level of the ribcage. The liver and other organs were gently shifted to expose the caudal surface of the diaphragm. The fascia underneath the diaphragm was carefully cut away. The insulated wire proximal to the exposed wire loop was sutured into position against the interior surface of the peritoneum just below the level of the diaphragm so that the uninsulated wire rested against the diaphragm itself. Contraction of the diaphragm consequent to stimulation, resulting in thoracic movement visually comparable with high amplitude breathing, was used to evaluate electrode placement. If stimulation failed to induce robust breathing, the suture was cut, and the electrodes were repositioned. Each electrode must be close to the point where the hemidiaphragm is innervated by the corresponding phrenic nerve; however, this point cannot be discerned visually. As a result, this mapping technique of positioning, stimulating, and repositioning the electrodes is necessary. A similar technique is used in humans to ensure proper electrode positioning in intraperitoneal diaphragmatic electrode implantation surgeries.^20^ Once effective electrode placement was achieved, the peritoneum was closed with absorbable suture. To further ensure that the location of the electrodes did not shift, the electrode wires were sutured against the exterior of the body wall close to the entry point into the peritoneum.

With the diaphragmatic electrodes fully implanted, the animal was placed in a prone position to expose the previously shaved scalp which was prepared with povidone-iodine, ethanol, and bupivacaine as described for the abdominal incision. An incision was made down the midline of the scalp. The animal was placed in a lateral recumbent position and the ends of the electrode wires were tunnelled under the skin, above the forelimbs, across the scapulae, and through the incision in the scalp. The remaining slack in the ∼8 cm wire was allowed to rest subcutaneously where it prevents movements of the animal’s head from applying tension on the electrodes against the diaphragm. The animal was returned to a supine position, and the abdominal incision in the skin was closed with absorbable suture leaving the electrode wire entirely under the skin with the exception of the terminal ends emerging from the incision in the scalp. The animal was then returned to a prone position and head fixed in a stereotaxic frame (Model 962; Koph, Tujunga, CA). The skull was exposed and cleaned with hydrogen peroxide (3% in distilled water) and allowed to dry. A scalpel was used to score the skull in a crosshatch pattern to facilitate dental cement adherence. Two bur holes were drilled (K.1070; Foredom Electric, Bethel, CT) in the skull and screw electrodes were threaded into these holes (E363/20; Plastics One). The female pins at the end of the diaphragm electrodes and the cortical screw electrodes were inserted into a 6-channel polyoxymethylene pedestal (MS363; Plastics One). The underside of the pedestal, electrode wires, screw electrodes, and the skull were adhered using dental cement forming a rounded headcap with the upper surface of the pedestal uncovered. The skin around the headcap was sutured closed, leaving only the pedestal exposed. While under anaesthesia, the animal was plugged into the diaphragmatic stimulation/EEG recording cable. The diaphragmatic pacing stimulation was applied while the animal was monitored visually to ensure that it displayed rhythmic thoracic movement comparable with rapid high amplitude breathing. Following the surgery, animals received 3.25 mg/kg buprenorphine (Ethiqa XR; Fidelis Pharmaceuticals, North Brunswick, NJ) s.c. as an analgesic and sterile saline (0.5–0.75 mL; Hospira, Lake Forest, IL) i.p. to facilitate recovery. Mice were allowed to recover for at least 1 week prior to experimentation. Adequate recovery was assessed by a lack of hunched posture and other signs of pain or distress; evidence of grooming behaviour; normal mobility and responsiveness to stimuli; and a lack of any signs of discolouration, swelling, or infection surrounding the scalp or abdominal incisions.

### Diaphragmatic pacing stimulation and EEG acquisition

Diaphragmatic pacing stimulation and EEG signal acquisition was conducted through different channels in the same six-channel cable (363–363; Plastics One). The EEG signal was relayed to a commutator (SL6C; Plastics One), amplified (FE232; ADInstruments, Sydney, Australia), digitized (PowerLab 16/35; AD Instruments) and passed to a personal computer where it was viewed live and recorded for subsequent analysis (LabChart; AD Instruments).

During diaphragmatic pacing, the diaphragm was stimulated via the two implanted loop electrodes (one on each hemidiaphragm, with the current passing between the two) with 150 ms trains of 1.8 mA, 50 Hz, biphasic square wave pulses. Because the stimulation was biphasic, which loop electrode was serving as the positive electrode and which electrode was serving as the negative electrode alternated with phase. Four stimulation trains were administered per second. The stimulation was generated with a dual output pulse stimulator (S88; Grass Instrument Co., West Warwick, RI) in conjunction with two analogue stimulus isolators (2200; A-M Systems, Sequim, WA).

### Whole body plethysmography

**Figure 1:**
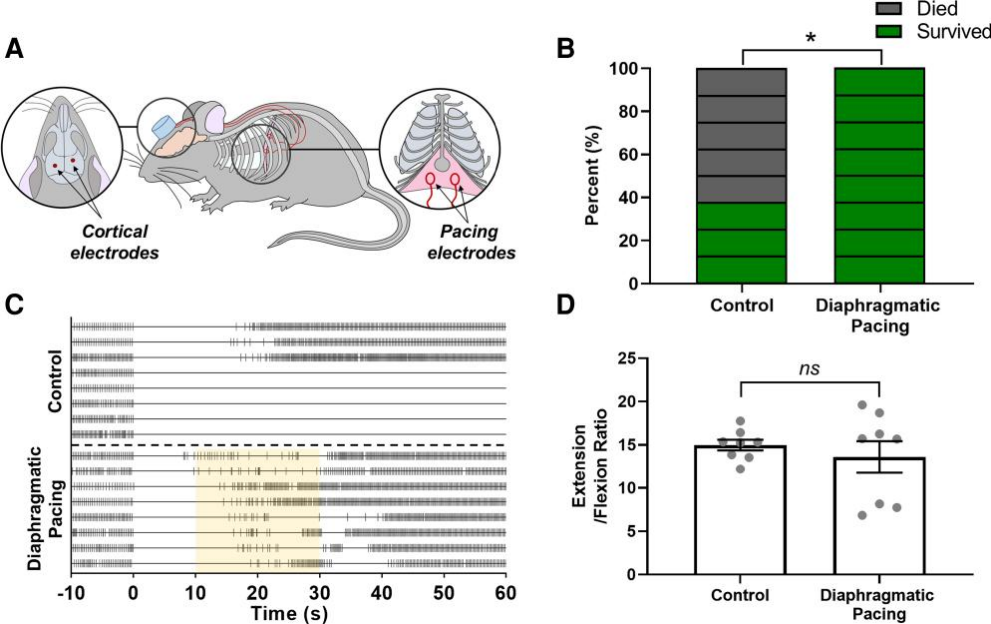
**Diaphragmatic pacing prevents seizure-induced death without altering seizure severity.** (**A**) Diagrammatic representation of the surgical instrumentation approach used in this study. Mice were equipped with epidural EEG electrodes and diaphragmatic pacing loop electrodes. The distal contacts of both electrodes were integrated into a headmount. (**B**) Percent mortality following control trials without diaphragmatic pacing (left, 62.5%) and experimental trials with diaphragmatic pacing (right, 0%; *P < 0.05, Fisher’s exact test; *n* = 8 mice). Each rectangle represents one mouse, the colour indicates the outcome of the experiment for that animal (green, survival; grey, death). (**C**) Raster plots depicting the timing of breaths before and after maximal electroshock seizure induction. Each horizontal line corresponds to an individual trial. Each vertical hash mark corresponds to a breath as detected by whole body plethysmography. The upper section depicts the eight control seizure trials, the lower section depicts the eight diaphragmatic pacing seizure trials. The yellow shaded area between 10 and 30 s following the seizure depicts the time in which the pacing stimulus was applied. (**D**) Motor seizure severity as measured by E/F ratio plotted as mean ± SEM (black lines) with individual values (grey circles; *ns*, *P* > 0.05; Unpaired two-tailed *t*-test; *n* = 8 mice).

**Figure 2:**
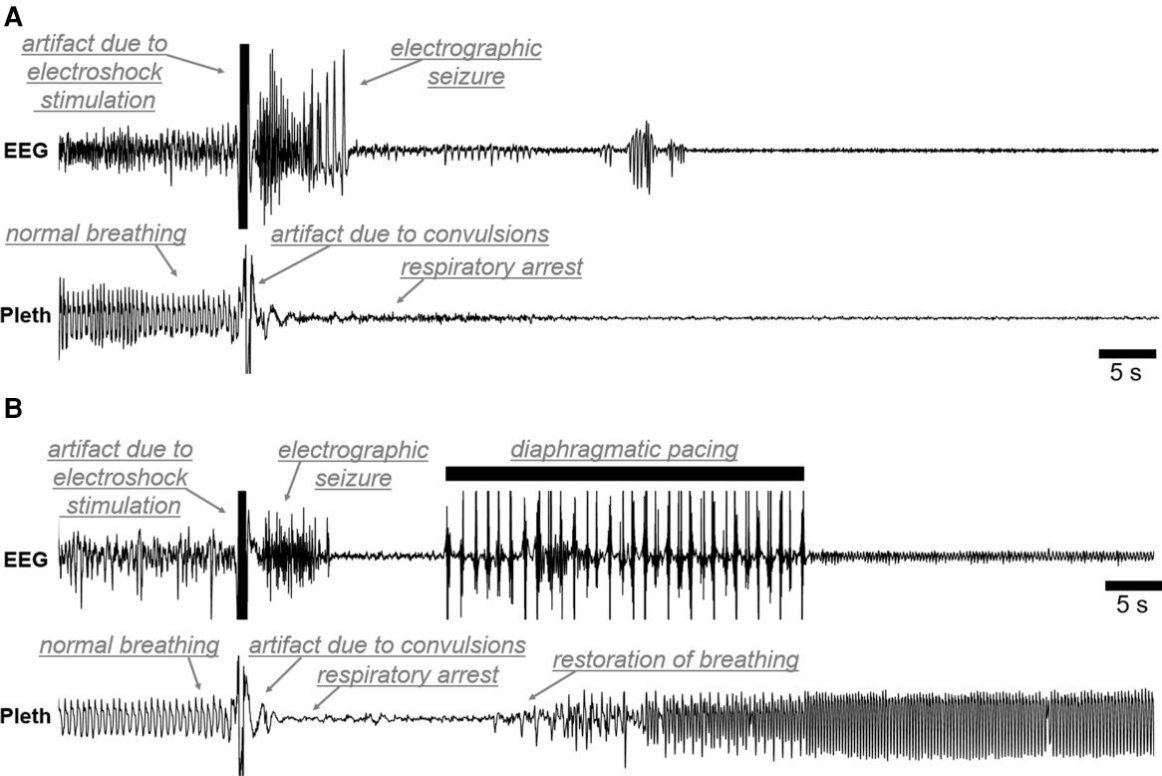
**Diaphragmatic pacing following the seizure prevents fatal respiratory arrest.** Representative EEG (upper) and plethysmography (lower) 60 s traces from a seizure trial without diaphragmatic pacing (**A**) and with diaphragmatic pacing (**B**). The animal which did not receive diaphragmatic pacing (**A**) died, the animal that did receive diaphragmatic pacing (**B**) survived. Notable features of each trace have been labelled (grey text). The diaphragmatic pacing stimulation caused pronounced artefacts on the EEG trace. Electrical artefact on the EEG trace due to the electroshock stimulation has been occluded (black bars) to prevent confusion with subsequent epileptiform discharges.

**Figure 3:**
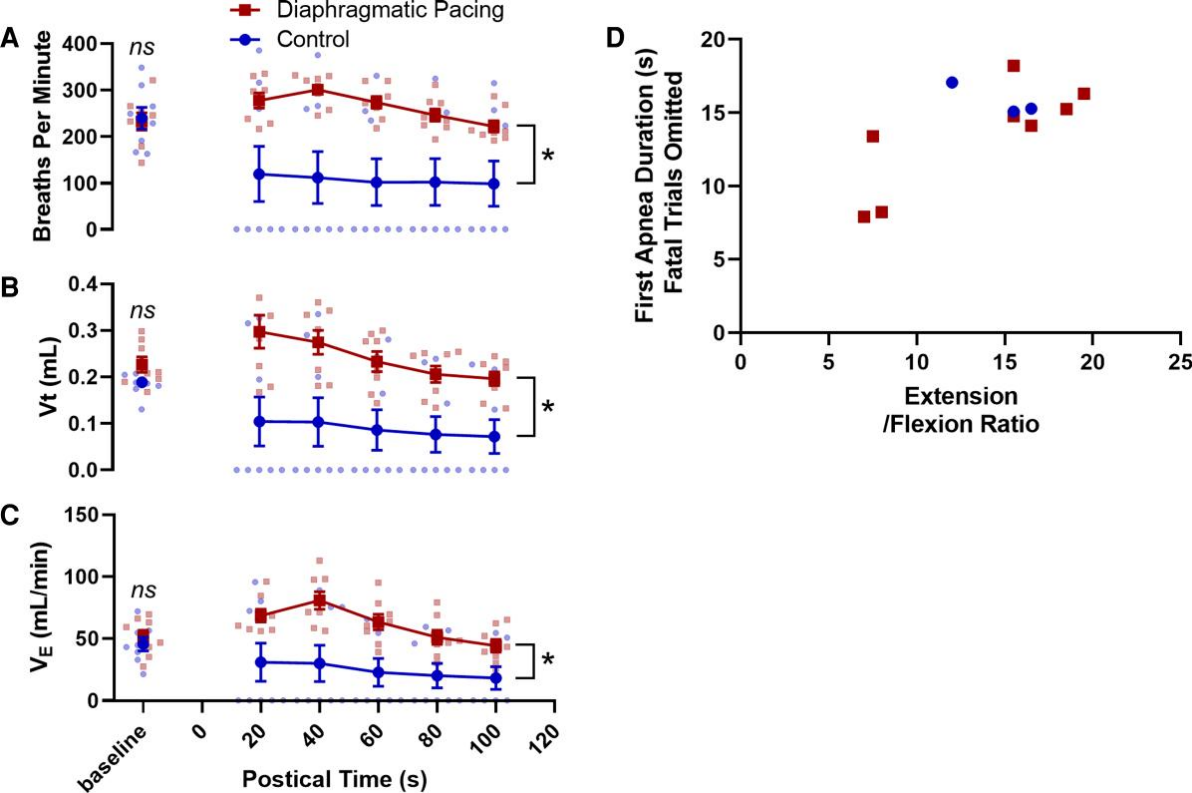
**Diaphragmatic pacing improved postictal breathing.** Time series data depicting preictal and postictal (**A**) respiratory rate in breaths per minute, (**B**) tidal volume in millilitres per breath, and (**C**) minute ventilation in millilitres per minute plotted as mean ± SEM with single data points for each mouse represented as blue or red dots (*ns*, *P* > 0.05, two-tailed *t*-test; *, *P* > 0.05, two-tailed two-way ANOVA; *n* = 8 mice per group, includes fatal and non-fatal trials). (**D**) Scatter plot depicting the relationship between duration of the first postictal apnoea and motor seizure severity as measured by the E/F ratio among animals that survived the seizure. Seizure severity was positively correlated with the duration of the first apnoea [r(10) = 0.73, *P* = 0.010].

Before maximal electroshock seizure trials, animals were removed from their home cage, set-up with the EEG tether, affixed with the maximal electroshock electrodes and placed in a cast acrylic plethysmography chamber (Data Sciences International, St. Paul, MN). Room air was drawn through the plethysmography chamber at 0.5 L/min. Baseline breathing was defined as the 30 s prior to seizure induction and postictal breathing was defined as the 120 s that followed seizure induction. Respiratory rate, tidal volume and minute ventilation were determined using a barometric flow-through method.^21^ Artefact-free segments of breathing were identified for analysis using the ‘Drorbaugh & Fenn Reduced Rejection’ algorithm with a 2 s log interval (601-1425-001; FinePointe, Data Sciences International).^21^ The ‘Drorbaugh & Fenn Reduced Rejection’ algorithm uses the size and frequency of preceding breaths to inform the identification of subsequent breaths. Because breaths in the initial period after the seizure are (i) not temporally proximal to the preceding breaths (Figs. 1C, 2B), (ii) irregular in their frequency (Figs. 1C, 2B) and (iii) of a low volume (Fig. 2B), the ‘Drorbaugh & Fenn Reduced Rejection’ algorithm is ill suited to the identification of breaths in the period immediately following the seizure. Therefore, for the purposes of identifying postictal breaths and determining apnoea duration, the timing of breaths was evaluated by a blinded experimenter (Figs. 1C, 3D). Furthermore, the initial 20 s after seizure induction was omitted from formal respiratory analysis (Fig. 3A–C) due to the motor convulsions during this time which generate plethysmographic artefacts that preclude accurate respiratory quantification and the high rate of algorithmic rejection during this time period. The percent of potential breaths rejected by the ‘Drorbaugh & Fenn Reduced Rejection’ algorithm for each respiratory epoch was as follows: baseline, 5.34%; 0–20 s, 64.12%; 20–40 s, 25.05%; 40–60 s, 1.07%; 60–80 s, 0.35%; 80–100 s, 2.07%; 100–120 s, 4.08%.

### Maximal electroshock seizures

A video camera recorded the animal’s behaviour within the transparent plethysmography chamber (C920S; Logitech, Lausanne, Switzerland). Video, EEG and baseline breathing data were recorded for 10 min prior to the seizure trial. After baseline data collection, the animal received a single electroshock stimulation (50 mA, 0.2 s, 60 Hz sine wave; Rodent Shocker; Harvard Apparatus, Cambridge, MA) via ear-clip electrodes wrapped with saline moistened gauze. Seizures were induced during wakefulness as assessed by live video. Seizure trials were conducted during the light phase between 1200 and 1800 clock time (between zeitgeber times 6 and 12).

Immediately at the onset of the stimulation the mouse takes a position analogous to a human ‘decorticate’ posture with the forelimbs rigidly pressed against the torso.^22^ This the ‘flexion’ phase of the motor seizure. This transitions into the ‘extension’ phase of the motor seizure in which both the forelimbs and hindlimbs become extended caudally towards the tail in a position analogous to a human ‘decerebrate’ posture.^22^ The extension phase of the motor seizure typically persists past the end of large amplitude cortical epileptiform discharges. Respiratory arrest begins early in the flexion phase, immediately after the stimulation. A subset of mice undergoing seizures induced with these parameters experience seizure-induced death.^23,24^ Non-fatal maximal electroshock seizures are characterized by a protracted initial apnoea followed by a series of smaller apnoeas. Fatal maximal electroshock seizures are characterized by a sustained terminal apnoea. Whole body plethysmography was used to quantify these apnoeas. The EEG trace is characterized by an initial high amplitude spike which is caused by the artefact from the stimulation current itself presumably along with massive neuronal depolarization followed by an electrographic seizure shortly thereafter.

The extension-to-flexion ratio (E/F ratio) was used to assess motor seizure severity.^25,26^ E/F ratio is computed by dividing the time the hindlimbs were flexed (extended <90° in relation to the torso) by the time that the hindlimbs were extended (projecting beyond 90° in relation to the torso) prior the relaxation of the body or the onset clonus. Antiseizure drugs abbreviate or eliminate the extension phase (resulting in an E/F ratio that is low or zero), this metric was the primary outcome measure for the maximal electroshock portion of the Anticonvulsant Screening Programme contracted by the National Institute of Neurological Disorders and Stroke.^27^

## Statistical analyses

All statistical analyses were completed using GraphPad Prism 7 (GraphPad Software Inc.) or Microsoft Excel. A two-tailed Fisher’s exact test was used to compare survival between conditions. An unpaired two-tailed Welch’s *t*-test was employed to compare E/F ratios between conditions, and an *F* test was used to compare E/F ratio variance. Unpaired two-tailed Student’s *t*-tests were used to compare baseline (30 s prior to seizure induction) respiratory rate, tidal volume and minute ventilation between experimental groups. Two-way ANOVAs were used to compare postictal breathing (20–120 s following seizure induction). The initial 20 s following seizure induction was omitted from respiratory quantification due to the occurrence of motor convulsions which generates plethysmography artefacts. A two-tailed Pearson’s correlation was used to compare the relationship between duration of the first apnoea and seizure severity. Unless otherwise specified, data are expressed as mean ± standard error. No animals or data were excluded from the study with the exception of the respiratory data from 0 to 20 s following the seizure (see the ‘Whole Body Plethysmography’ subsection for more details). All data was scored by an experimenter blinded to the treatment condition of the animal. The sample sizes of groups were determined using pilot data in the lab. All data was analyzed by an experimenter blinded to experimental condition. Significance threshold was set at *P* < 0.05 for all comparisons.

## Data availability

The data that support the findings of this study are available from the corresponding author, upon reasonable request.

## Results

### Experimental design

Mice were surgically instrumented with EEG and diaphragmatic pacing electrodes (Fig. 1A). Following surgical recovery, maximal electroshock seizures were induced. Mortality (Fig. 1B, C) and motor seizure severity (Fig. 1D) were assessed. EEG (Fig. 2) and plethysmography (Figs. 2,3) recordings were made during the seizure trials. In the experimental group, the diaphragm was stimulated to exogenously induce breathing between 10 and 30 s following seizure induction (Fig. 1C, lower). In the control group, no stimulation was applied to the pacing electrodes and seizure-induced respiratory arrest was allowed to proceed unchecked (Fig. 1C, upper). We predicted that diaphragmatic pacing would protect against seizure-induced death.

Experimenters were blind to the group of the animal until the time of the trial at which point animals were assigned to groups with a ‘simple randomization’ approach until one group reached the intended sample size (*n* = 8 mice, two groups, total of 16) at which point the remaining animals were used for the other group.

### Diaphragmatic pacing prevented seizure-induced death but did not alter seizure severity

Mortality in the control group (5/8, 62.5%) was comparable with that reported in wild-type male mice in prior investigations.^4–9^ Comparison of mortality rates between the control group and the diaphragmatic pacing group (0/8, 0%) using a 2 × 2 two-tailed Fisher’s exact test revealed a statistically significant reduction in seizure-induced death (*P* = 0.026, *n* = 8 mice; Fig. 1B).

Mean motor seizure severity, as assessed by the E/F ratio, in the diaphragmatic pacing group (*M* = 13.59, *SD* = 5.18) was not different from the control group (*M* = 14.97, *SD* = 1.74) when compared with a unpaired two-tailed *t*-test with Welch’s correction [*t*(8.56) = 0.72, *P* = 0.493; Fig. 1D]; however, the diaphragmatic pacing group had higher variance in E/F ratios (*F*_7,7_ = 8.85, *P* = 0.010, *n* = 8 mice). This increase in variance may be the result of difficulties in distinguishing between motion associated with the pacing stimulation and motion associated with relaxation of the animal’s body or the onset of clonus. The end of the extension phase is assessed by the onset of clonic convulsions or relaxation of the torso/limbs, before this point the animals body appears rigid and almost perfectly still. The diaphragmatic pacing stimulation may have generated slight motion in the torso even if no air was being exchanged due to airway occlusion. Thus, it is conceivable that this motion might have complicated the scoring of the end of the extension phase which was happening at or around the same time. It may be that duration of the first apnoea is related to an electrographic phenomenon resulting in central or obstructive apnoea which is correlated to the progression of the motor phenotypes seen in maximal electroshock seizures.

### Diaphragmatic pacing improved postictal respiratory outcomes

Unpaired two-tailed *t*-tests identified no differences between the control and experimental groups in preictal respiratory rate [*t*(14) = 0.24, *P* = 0.811, *n* = 8 mice; Fig. 3A], tidal volume [*t*(14) = 1.91, *P* = 0.077; Fig. 3B] and minute ventilation [*t*(14) = 0.74, *P* = 0.982; Fig. 3C]. Two-way ANOVAs revealed statistically significant associations between diaphragmatic pacing treatment and postictal respiratory rate (*F*_1,84_ = 37.46, *P* < 0.001, *n* = 8 mice, η^2^ = 0.28; Fig. 3A), tidal volume (*F*_1,84_ = 47.61, *P* < 0.001, η^2^ = 0.32; Fig. 3B) and minute ventilation (*F*_1,84_ = 36.78, *P* < 0.001, η^2^ = 0.27; Fig. 3C). Among animals that survived the seizure, seizure severity was positively correlated with the duration of the first apnoea [*r*(10) = 0.73, *P* = 0.010, *n* = 11 mice, R^2^ = 0.54; Fig. 3D]. Unexpectedly, resumption of breathing was not instantaneous at the onset of diaphragmatic pacing. On average, there was a 3.52 s latency between the onset of the pacing stimulus and the first breath (*SD* =  ± 1.30 s, Figs. 1C, 2B); However, two animals took their first breath just prior to the onset of the pacing stimulation. Furthermore, although all mice breathed at some point during the pacing stimulation, most animals breathing was not eupnoeic during or following the stimulation.

## Discussion

SUDEP is the leading cause of epilepsy related death.^1^ It is generally agreed that central respiratory failure plays a crucial role in the pathophysiology of SUDEP.^3,4^ Evidence from human patients and animal models indicates that ventilatory assistance during the postictal period may prevent seizure-induced death.^3–29^ Diaphragmatic pacing is an FDA approved surgical intervention in which electrodes on the diaphragm or phrenic nerve(s) are stimulated to provide ventilatory assistance to patients who would otherwise undergo central respiratory arrest in conditions such as spinal cord injury, central hypoventilation syndrome, and central sleep apnoea.^12,17–30^ The goal of this study was to test the hypothesis that seizure-induced respiratory arrest and death can be prevented with transient diaphragmatic pacing. To test this hypothesis, we implanted mice with EEG and diaphragmatic pacing electrodes and induced seizures using maximal electroshock (Fig. 1A). In the experimental group, diaphragmatic pacing was applied between 10 and 30 s following the seizure (Figs. 1C, 2B). In the control group, no diaphragmatic pacing stimulus was applied (Figs. 1C, 2A). We observed that diaphragmatic pacing treatment prevented seizure-induced respiratory arrest and death (Fig. 1B) and improved postictal respiratory outcomes (Fig. 3A–C). The findings of this investigation may inform the implementation of diaphragmatic pacing strategies for the prevention of SUDEP.

In this study, seizures were electrically induced by the experimenter and diaphragmatic pacing was performed during a fixed time interval; however, in epilepsy patients, seizures are spontaneous and SUDEP typically happens while the patient is alone meaning that there would be no one to assess the patient’s breathing and initiate diaphragmatic pacing.^31^ As a result, clinical implementation of diaphragmatic pacing for the prevention of SUDEP would likely necessitate a closed-loop control system capable of (i) passively monitoring the patients breathing, (ii) recognizing that the patient has stopped breathing, (iii) stimulating to provide ventilatory assistance and (iv) discontinuing the pacing stimulation when it was no longer necessary. Fortunately, a device with the these characteristics already exists and has been implemented in the context of central sleep apnoea.^17^ The Remedē System (Respicardia, Minnetonka, MN) (i) records impedance from an electrode the left pericardiophrenic, right brachiocephalic or azygos vein to monitor breathing; (ii) integrates the respiratory data with time of day, activity level and body position to determine whether stimulation is necessary; (iii) provides transvenous stimulation of the phrenic nerve using the electrode in the pericardiophrenic or brachiocephalic vein to elicit breathing and (iv) uses the patient’s body position, activity and endogenous respiration to determine when stimulation can be discontinued.^17^ Extant seizure detection devices which alarm in response to seizure activity are ailed by false positives which are disruptive to the patient, particularly when they occur during sleep.^32^ By contrast, transvenous stimulation of the phrenic nerve is sufficiently unobtrusive as to not disrupt sleep.^17^ Thus, if this device could be applied to prevent seizure-induced respiratory arrest, a false positive stimulation would probably not wake up the patient whereas a true positive would prevent the patient from never waking up.

Although it is not possible to prospectively identify individuals will undergo SUDEP, certain epilepsy subtypes are drastically more likely to elicit SUDEP. Dravet syndrome is an epileptiform encephalopathy caused by mutations in the gene *SCN1A.*^33^ Dravet syndrome is notorious for high rates of SUDEP^34^ potentially making it well suited to this intervention. Furthermore, though SUDEP is an uncommon occurrence in epilepsy patients, non-fatal respiratory disruption is a common feature of convulsive seizures.^35^ These respiratory disturbances and the associated derangement in blood gasses may contribute to a variety of adverse outcomes such as neurodegeneration, postictal generalized EEG suppression, and seizure-induced memory impairments.^36,37,38^ Thus, diaphragmatic pacing to counteract seizure-induced respiratory disruption may have benefits for patient health beyond the prevention of SUDEP.

The barrier to clinical translation of diaphragmatic pacing for SUDEP prevention may be eased by the use of responsive neural stimulators, deep-brain stimulators and vagal nerve stimulators in epilepsy patients.^39,40,41^ It is conceivable that these devices could be augmented to include diaphragmatic pacing functionality. This is particularly true for vagal nerve stimulators as the vagal and phrenic nerves are so close (<2 cm in humans^42^) that vagal nerve stimulators can depolarize the phrenic nerve inadvertently causing contraction of the diaphragm.^43^

It was our expectation that robust breathing would resume at the onset of the diaphragmatic pacing stimulation. This was not the case, there was a 3.52 s delay on average between the start of pacing and the first postictal breath (Figs. 1C, 2B). One possibility is that seizure-induced laryngospasm occluded the airway during the early part of the pacing stimulation. Periictal laryngospasm has been demonstrated during seizures in several animal models.^44,45,46^ Seizure-induced laryngospasm has been observed in epilepsy patients;^47^ however, methodological challenges inherent to assessing laryngospasm in seizing humans has made it difficult to determine its prevalence. Seizure-induced laryngospasm has been proposed as a contributing mechanism in the pathophysiology of SUDEP.^46,47^ Investigations using endotracheal implants in the DBA/2 model of seizure-induced death have implicated laryngospasm as a contributing factor to mortality.^48^ A similar approach using endotracheal implants may be useful in determining if laryngospasm is responsible for preventing immediate restoration of breathing during postictal diaphragmatic pacing in maximal electroshock. Future investigations should also attempt to implement a closed-loop diaphragmatic pacing system to prevent seizure-induced death in an animal model with spontaneous seizure-induced death such as the Scn1a^R1407X/+^ or Kv1.1 null mouse lines which better recapitulate the characteristics SUDEP.^49,50^ Maximal electroshock was used in this study because it features seizure-induced respiratory arrest the timing of which can be precisely controlled by the experimenter. Maximal electroshock is a common method for studying the potential underlying mechanisms of SUDEP;^7–23,4–8^ However, it does not recapitulate some potentially significant aspects of clinically observed SUDEP. In maximal electroshock seizures respiratory arrest begins during the stimulation and persists into the postictal period whereas, in SUDEP, the fatal apnoea typically emerges during the postictal period.^3^

With regards to the timing of the first postictal breath in the diaphragmatic pacing group, it should also be noted that two animals took their first breath prior to the onset of the pacing stimulation (Fig. 1C). Presumably these animals, and potentially others in the diaphragmatic pacing group, would not have died of seizure-induced respiratory arrest regardless of the pacing stimulation given that maximal electroshock seizures do not always have a fatal outcome (Fig. 1B, C).^7,28–51^ Furthermore, though all mice breathed at some point during the pacing stimulation, most animals breathing was not eupnoeic following the stimulation. Whether the lack of eupnoeic breathing following the diaphragmatic pacing stimulation is the result of central apnoea, laryngospasm or some other mechanism is currently unclear and warrants further investigation.

Currently, we are unable to prevent SUDEP.^52,53^ We are unable to identify those who are at the greatest risk of dying of SUDEP.^54^ It is not entirely clear why some seizures result in fatal respiratory arrest when the overwhelming majority do not.^4^ The results of this study suggest that diaphragmatic pacing, an FDA approved treatment which exogenously induces ventilation, may be capable of preventing death following this devastating and highly enigmatic phenomenon.^55^

## Abbreviations

FDA =: United States Food and Drug Administration
SUDEP =: sudden unexpected death in epilepsy
